# A Novel Method for Long-Term Analysis of Lactic Acid and Ammonium Production in Non-growing *Lactococcus lactis* Reveals Pre-culture and Strain Dependence

**DOI:** 10.3389/fbioe.2020.580090

**Published:** 2020-10-08

**Authors:** Avis Dwi Wahyu Nugroho, Michiel Kleerebezem, Herwig Bachmann

**Affiliations:** ^1^TiFN, Wageningen, Netherlands; ^2^Health Department, NIZO Food Research, Ede, Netherlands; ^3^Laboratory of Host-Microbe Interactomics, Wageningen University and Research Centers, Wageningen, Netherlands; ^4^Systems Biology Lab, Vrije Universiteit Amsterdam, Amsterdam, Netherlands

**Keywords:** high throughput screening, long-term biocatalysis, conversion decay, prolonged acidification, arginine utilization, lactic acid bacteria

## Abstract

In various (industrial) conditions, cells are in a non-growing but metabolically active state in which *de novo* protein synthesis capacity is limited. The production of a metabolite by such non-growing cells is dependent on the cellular condition and enzyme activities, such as the amount, stability, and degradation of the enzyme(s). For industrial fermentations in which the metabolites of interest are mainly formed after cells enter the stationary phase, the investigation of prolonged metabolite production is of great importance. However, current batch model systems do not allow prolonged measurements due to metabolite accumulation driving product-inhibition. Here we developed a protocol that allows high-throughput metabolic measurements to be followed in real-time over extended periods (weeks). As a validation model, sugar utilization and arginine consumption by a low density of translationally blocked *Lactococcus lactis* was designed in a defined medium. In this system *L. lactis* MG1363 was compared with its derivative HB60, a strain described to achieve higher metabolic yield through a shift toward heterofermentative metabolism. The results showed that in a non-growing state HB60 is able to utilize more arginine than MG1363, and for both strains the decay of the measured activities were dependent on pre-culture conditions. During the first 5 days of monitoring a ∼25-fold decrease in acidification rate was found for strain HB60 as compared to a ∼20-fold decrease for strain MG1363. Such measurements are relevant for the understanding of microbial metabolism and for optimizing applications in which cells are frequently exposed to long-term suboptimal conditions, such as microbial cell factories, fermentation ripening, and storage survival.

## Introduction

The widespread use of bacteria in many biotechnological applications is not only attributed to their growth ability but also to their metabolic persistence under non-growing/dormant condition. The arrest of cell division coincides with limited de-novo protein synthesis, whereas metabolic activity and survival can be maintained over a long period of time (Ercan et al., 2015; Erkus et al., 2016). This non-growing state can be induced by adverse circumstances, e.g., starvation, lethal stress or inhibitory compounds (Oliver et al., 1995; Keren et al., 2004; Magajna and Schraft, 2015), as commonly found in industrial processes, such as bioreactor metabolite production (Förberg et al., 1983), wastewater treatment (Witzig et al., 2002), and food processes (Millet and Lonvaud-Funel, 2000). In some applications, for example microbial cell factories, such physiological state might be desired since metabolic fluxes are diverted away from cell growth, resulting in the increase of metabolic yield (Sonderegger et al., 2005). In food fermentation applications, the long-term metabolic activity is an important function of starter cultures that contributes to product quality, stability and safety (Marcobal et al., 2006; Liu et al., 2018). Therefore, the study of persisting metabolic activity in non-growing cells is of relevance for food fermentation processes, and the ability to steer the activity of such cells can strongly contribute to process control.

Lactic acid bacteria (LAB) and *Lactococcus lactis* in particular are important industrial microorganisms and the ability to steer their metabolic activities in non-growing cultures is of great interest. As an example, the majority of flavor volatiles is produced by the starter culture during cheese ripening, where the most relevant ones are derived from nitrogen catabolism, such as 3-methylbutanal, 2-methylbutanal, and 2-methylpropanal (Smid and Kleerebezem, 2014). The combination of cheese production-related environments, such as the dynamic processing conditions, low temperatures, high salt concentrations, and carbon starvation results in non-growing but metabolically active starter culture cells. Despite the loss of culturability during cheese ripening, it has been shown that a high level of cellular intactness was retained and only a small fraction of the starter population appeared to exhibit membrane injury (Erkus et al., 2016). Furthermore, studies on the metabolite production of *Lactococcus lactis* have shown that the cheese-related volatile profile can be mimicked best with near-zero growth conditions achieved by retentostat cultivation (van Mastrigt et al., 2018b) or by incubation in nutrient-free buffer (van de Bunt et al., 2014). Conversely, the prolonged metabolic activity of non-growing starter cultures can also pose challenges in ensuring product quality during storage. Post-production acidification is an important example of such phenomenon, in which slow but persistent lactic acid production in stored yogurts eventually leads to shorter shelf life due to perceivable changes in flavor and acidity leading to lower consumer appreciation and acceptance (Shah et al., 1995; Settachaimongkon et al., 2016).

Since the renewal of proteins is limited, the role of repair, maintenance and active degradation becomes particularly important. During prolonged incubation of non-growing cells, enzyme decay is inevitable but can be minimized by intracellular mechanisms that ensure protein quality control, such as the Clp-related protease machinery (Frees et al., 2001; Kock et al., 2004). Upon carbon starvation, lactococci were shown to lose the ability to be cultured but at the same time they could maintain intact membranes and showed metabolic activity for up to 3.5 years (Ganesan et al., 2007). The overall cell fitness and performance can be greatly affected by the cellular proteome composition that is dependent on the conditions applied during the growing phase (Dijkstra et al., 2014; Settachaimongkon et al., 2015). Consequently, the initial enzyme amount and the rate of enzyme-activity decay will influence the overall production of metabolites over prolonged periods of incubation. In addition, environmental conditions, such as pH, and temperature, as well as the level of oxidative conditions, and inhibitor exposure also affect protein and cellular stability. Collectively, these environmental parameters determine the actual rate and stability of metabolite formation, but they are often determined as separate entities and/or in a simplified *in vitro* system.

In this study, we developed a method that allows us to measure catalytic activity of a complete pathway based on continuous pH monitoring through a fluorescent readout in a 384-well plate format. pH-monitoring is employed as a model since it applies to primary metabolism but also amino acid catabolism, that both involve a substantial metabolic flux. The system is designed to prevent product inhibition, which is achieved by employing cultures at low cell densities combined with the translation-inhibiting antibiotic erythromycin. Blockage of de-novo protein synthesis enabled us to compare the impact on acidification and arginine consumption rate of distinct strains and cellular proteome compositions, that were generated through different (pre)-culture conditions. The method allowed continuous pH measurements in real-time over periods of several weeks, without the emergence of detectable product inhibition as commonly found in batch systems.

## Materials and Methods

### Strain and Cultivation Conditions

*Lactococcus lactis* subsp. *cremoris* NCDO712 (Gasson, 1983), HB60 (Bachmann et al., 2013), and MG1363 (Wegmann et al., 2007) were grown on chemically defined medium for prolonged cultivation (CDMPC) as described previously (Price et al., 2019). Medium was supplemented with either lactose 30 mM, galactose 55 mM, or glucose 55 mM depending on experimental design. All pre-cultures were standardized and started with a single use aliquot of glycerol stock which was 1,000-fold diluted in medium and cultured overnight (as described in Price et al., 2019). Overnight cultures were sub-cultured (40-fold dilution) in fresh medium and harvested during the early exponential phase (OD 0.1–0.2). *Lactococcus lactis* was routinely cultured at 30°C without aeration. The growth rate of *L. lactis* NCDO712 in CDM+0.5% glucose is approximately 0.7/h, which is a bit slower compared to 0.8/h reached in the rich medium M17 supplemented with 0.5% glucose.

### Prolonged Measurements of Culture pH

Exponentially growing cells were centrifuged at 5,000 g for 10 min and washed twice with an equal volume of PBS, followed by resuspending the cells at a standard density between 1E+07 and 2.5E+07 cells/mL in fresh CDMPC (Mn-omitted) supplemented with erythromycin (5 μg/mL) and 10μM 5(6)-carboxyfluorescein (Sigma-Aldrich 21877). Individual sugar was supplemented as the sole carbon source in the media depending on experimental design at concentration of 30 mM (lactose) or 55 mM (glucose or galactose). In experiments measuring arginine utilization, the CDMPC pH was set to 5.5 (rather than 6.5 in standard CDMPC) and instead of one of the sugars, L-arginine was supplemented at a final concentration of 30 mM. All measurements were performed with at least four replicates in black clear bottom 384-well plate (Greiner Bio-One 781076). Fluorescence (λex/em: 485/535 nm) was measured at constant gain, at 30 min intervals during a period of up to 3 weeks at 30°C in a microplate reader (Tecan Safire 2). The gain was determined to ensure standard pH solutions (4.0–7.0) were in the detectable range.

### Viable Count and Membrane Integrity Determination

Measurements of culturable bacteria were performed through plating on CDMPC supplemented with 1% glucose and 0.5% UltraPure agarose (Invitrogen 16500500). Serial dilutions were prepared in PBS and 100 μL of the diluted cultures were plated on agar plates. Plates were incubated at 30°C for 24–48 h and colonies were enumerated.

Membrane integrity of cells during prolonged incubation was analyzed using Live/Dead BacLightTM Bacterial viability and counting kit (Invitrogen L34856) and a BD LSR Fortessa Flow Cytometry instrument (BD Biosciences), according to manufacturer instructions with some modifications. A staining mixture was prepared with 1.5 μL of PI, 1.5 μL of SYTO 9 stock-solutions, 5 μL microsphere standard (1E+08 beads/mL), 892 μL of running buffer (FACS Flow), and 100 μL of sample resulting in a total of 1 mL assay reaction. Fluorescence signals were measured with FITC and PE-Texas Red detectors. Gating was set on the basis of fresh overnight culture (live) and cells incubated in 60% ethanol (dead).

### Fermentation End Product Analysis

The concentrations of organic acids in media (lactic acid, formic acid, acetic acid, and ethanol) were determined by high performance anion exchange chromatography (HPAEC) with UV and refractive index (RI) detection as previously described (Hugenholtz and Starrenburg, 1992). Culture supernatant samples were collected and filtered using 0.20 μm polyethersulfone (PES) membranes and stored at -20°C before analysis.

### Calculation of Lactic Acid or Ammonia Production Rate

Raw data files from the microplate reader were analyzed and plotted with R (v 3.6.1). Fluorescent signals were converted to pH values based on a standard curve obtained with fluorophore-containing medium set at a range of pH values. To correct for buffering capacity, the proton equivalent of the pH values were calculated. Subsequently, the acid production was determined based on the logarithmic equation which relates the accumulation of acid and the change in proton equivalent. This relation was obtained from a titration curve which was prepared by step-wise addition of lactic acid (2 M) to CDMPC in the presence of 2.5E+07 cells/mL for the extended operational range of the measurement (pH 6.5–4.0). In case of arginine consumption, the ammonia production was analogously determined using a titration curve of ammonia from pH 5.5–7.0. The production rate (M/h) was calculated periodically in equal intervals of lactate or ammonium production e.g., every 0.001 M.

## Results

### Optimization of Cell Density and Medium Composition Allows Acidification Measurements for Weeks Without Product Inhibition in Non-growing Cells

The glycolytic flux of *Lactococcus lactis* has been reported to run at maximal rate during balanced growth in batch culture (Koebmann et al., 2002b). The high flux through glycolysis and lactic acid production leads to a fast decline in pH and high accumulation of lactate, which eventually stops acidification. In non-growing cells of *L. lactis*, the glycolytic flux was found to be 37% of exponentially growing cells (Koebmann et al., 2002a), which is still relatively high. To enable a prolonged measurement of the product formation rate, a number of challenges, such as product inhibition, continuous monitoring, sufficient throughput, and the maintenance of non-growing state need to be overcome. To achieve this, we established a microplate-based assay in which the pH of non-growing cells can be continuously followed in a microplate reader through the use of commercially available fluorescent pH indicator (5/6-carboxyfluorescein). The assay consists of a defined medium in which cells are fully translationally blocked with erythromycin (5 μg/mL) and provided with excess supply of a catabolizable substrate. Under circumstances with e.g., sugar (1% w/v) as a substrate and high cell densities (e.g., 1E+08 cells/mL), this setup leads to full acidification of the solution within 24–48 h, when further acidification is blocked by product inhibition. In contrast, in the absence of translation inhibition, the exponential growth of cells and the concomitant increase of glycolytic flux will typically lead to complete acidification within less than 8–12 h. Therefore, to enable long term acidification online monitoring, we ensured the cell density is kept constant by complete inhibition of protein translation.

**FIGURE 1:**
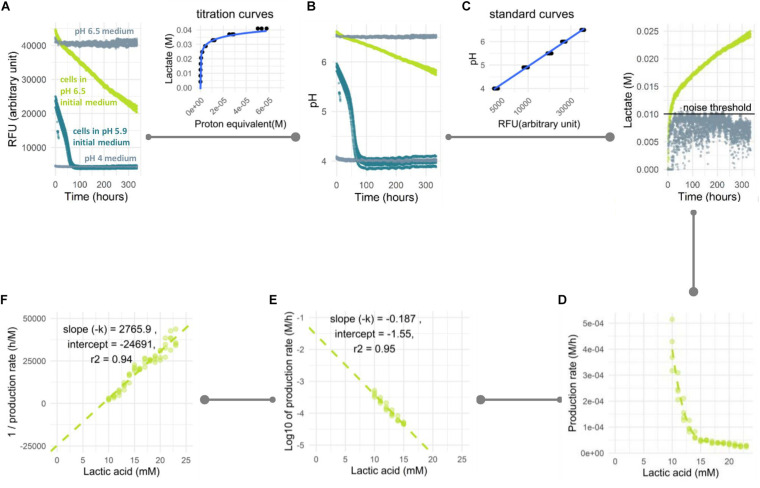
Analysis pipeline of prolonged acidification in non-growing cells. *L. lactis* NCDO712 cells were pre-cultured in lactose 1%, harvested at mid-exponential growth and transferred to the assay medium with lactose 1%. **(A)** Fluorescent signal was measured for assay medium without cells at pH 4 and 6.5 (light gray), cells (2.5E+07/mL) in assay medium at an initial pH of 6.5 (light green) and an initial pH of 5.9 (dark green). **(B)** Based on a standard curve, the RFU were converted to pH. In samples with a low starting pH (dark green) acidification stopped due to product inhibition at pH 4 while for a high starting pH acidification was still ongoing after more than 300 h. **(C)** Based on a titration curve, the pH profile was converted to the amount of lactic acid produced. The noise threshold was determined from the non-biological fluctuation of signal based on a negative control at pH 6.5. **(D)** The lactate production rate is plotted as a function of the total amount of lactic acid produced. Decline in production could be calculated following first order **(E)** or second order **(F)** reaction kinetics. The experiment was carried out three times with four replicates with reproducible results. Four replicate curves are shown in this plot.

Cell concentration in the range of 1–2.5E+07/mL in 20 mM phosphate buffer resulted in slow but detectable acidification activity that remains in a high-buffering range. This allowed us to follow measurements for 3 weeks. The results consistently showed that acidification ranged in pH from 6.5 to 5.75 and produced less than 20 mM lactate over the complete period. Considering the pKa of lactate at 3.8, the majority of lactate (∼99%) will be in a deprotonated state, which will not readily diffuse across the cell membrane. During the long period of measurement, the fluorescent signal was stable and showed negligible change in signal intensity (Figure 1A). The detection accuracy and frequent reading interval (30 min) allows real-time and precise monitoring. We opted to use a defined medium as it allows well-defined alterations of individual constituents, however, it is possible to use undefined medium, such as M17, but medium-composition manipulations will be less defined and higher fluorescence background might reduce measurement resolution. Furthermore, the use of assay medium that closely resembles growing medium of the bacteria aims to optimize conditions for all cellular processes. In combination with the use of a microplate (384-wells), the setup allows high-throughput comparisons of prolonged acidification profiles.

### Conversion of the Fluorescent Signal to Acid Production Rate

The fluorescent signal in our assay was not directly reflecting organic acid production because of the buffer capacity being higher at the initial (near neutral) pH compared to the buffering capacity toward the end of the fermentation (>pH 5). To get a better representation of lactic acid production, we established an analysis pipeline where the fluorescent signal (Figure 1A) is first converted to pH (Figure 1B) based on a calibration curve of medium prepared at different pH values (pH was set by hydrochloric acid addition). Subsequently, the pH value is converted to the proton equivalent, which is then used to obtain the equivalent of acid produced (Figure 1C). The latter conversion was based on the titration of pure lactic acid to the assay medium in the presence of cells to accommodate the contribution of cells to buffering capacity. Once the acid formation over time was calculated, the rate of formation and its decline could be derived as a function of time. However, large differences in rate could result in unfair comparison due to the difference in total reaction number per enzyme. Therefore, the production rate was calculated within periods of equal production of acid e.g., 1 mM starting from the period above the noise threshold determined from a negative control (Figure 1C). In multiple experiments, the signal considered as noise was consistently found to be the first 7.5–10 mM of lactate produced. This can be seen in the negative control that showed signal noise at 5–10 mM of lactate produced (Figure 1C). This sensitivity to noise is possibly due to the high buffering capacity in this pH region, which leads to small signal variation resulting in relatively large noise in lactate production (with a relatively constant signal of approximately 40,000 RFU in the control sample (Figure 1A) noise of 1,000–1,500 RFU is responsible for the observed 5–10 mM variation in lactate). The measurements from this initial region were therefore not considered in the data analysis. The decline in lactic acid production rate over time was plotted against lactate accumulation (Figure 1D) and typically resulted in a straight-line when transformed to its log-values (Figure 1E) or its inverse (Figure 1F), following the first order or second order of rate kinetic, respectively. From this plot, the kinetic of rate decline could be determined from the slope and the maximum/initial production rate was predicted from Y-intercept. Based on these kinetic parameters, the behavior in longer period of time could be estimated, e.g., lactate yield in 2 months. As an exemplary case, *L. lactis* NCDO712 (2.5E+07 cells/mL) pre-cultured in lactose was transferred into the assay medium containing the same sugar and followed for 2 weeks. A 15-fold reduction of the lactic acid production rate from 3.98E-04 to 2.51E-05 M/h was observed (Figure 1D).

### Translational Blocking Leads to Non-culturable Cells and Has No Influence on Organic Acid Profiles

**FIGURE 2:**
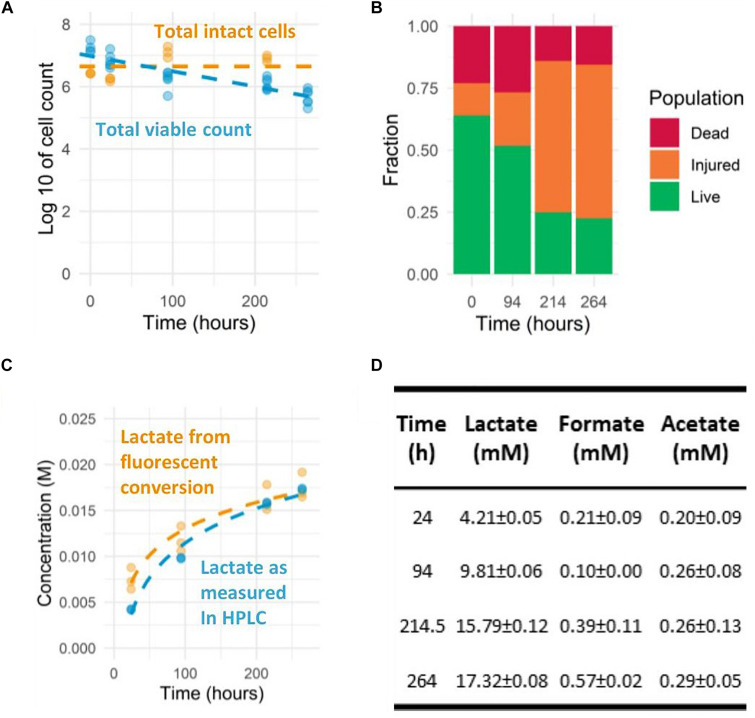
Prolonged observation of *L. lactis* NCDO712 pre-cultured in CDMpc-lactose 1%, harvested at mid-exponential growth and transferred to the assay medium with lactose 1%. **(A)** Colony forming units and intact cell count as measured by flow-cytometry. **(B)** The different cell fractions of the life-dead staining as measured by flow-cytometry. **(C)** Lactate accumulation derived from fluorescent measurements and HPLC determination show good agreement (R-squared value of 0.94) (dashed lines indicate semi-logarithmic fit). **(D)** The organic acid production profiles as determined by HPLC were dominated by lactic acid throughout the experiment.

The addition of erythromycin results in the physical blocking of the nascent-peptide exit tunnel in the ribosome which halts translation (Tenson et al., 2003). We tested different erythromycin concentrations and found that 5 μg/mL was sufficient to prevent an increase in culture density over 2 weeks, indicating that cell growth was blocked due to continuous translational blocking. Such treatment may cause cell dormancy or induce cell death which is influenced by not only the kinetics of drug-ribosome interactions, but also species or strains, growth conditions, cell density, and the antibiotic concentration (Svetlov et al., 2017). When prolonged translation inhibition is applied, cell death is inevitable and can be responsible to the decline in product formation to some extent. Antibiotic exposure to *Lactococcus lactis* has also been reported to induce heterogenous population response regarding dormancy states and the corresponding death rates and metabolic activity (van Tatenhove-Pel et al., 2019). To characterize the effects of erythromycin in our experiments, a combination of CFU counting and live-dead staining measurements was employed to determine how cellular viability and integrity relate to the observed decline in acidification. For *L. lactis* NCDO712 pre-cultured and transitioned to lactose, the amount of colony forming units was decreasing from 7.25 to 5.65 log10 CFU/mL in 11 days (Figure 2A), which is equivalent to roughly a 40-fold overall decline. On the other hand, the number of intact cells according to live-dead stained flow cytometry counts was more or less constant at approximately 7 log cells/mL. Moreover, the fraction of cells classified as intact (live+injured-cells) remained constantly above 75% (Figure 2B), displaying a slow progression toward cell populations with compromised membrane-integrity (“injured cells” in the analysis). In the 2-week incubation period, approximately 40% of the cells initially qualified as “live” cells progressed to the “injured” population, representing only a 2.5-fold reduction of the “live” population, which is much less than the observed 40-fold reduction in CFU enumerations. This observation is in agreement with the detection of so-called “viable but non-culturable” (VBNC) *L. lactis* cells during 2 weeks of retentostat cultivation (van Mastrigt et al., 2018a) and cheese ripening (Erkus et al., 2016). Overall, the 40-fold decrease in CFU does not match the 15-fold decrease in acidification rate and the results of the live-dead staining. This suggests that VBNC cells appear in the population, but the current methodology does not allow to discern the contribution of different cell populations to the acidification profiles.

Besides cell viability, we also determined the concentration of organic acids during the prolonged acidification measurements to confirm the fluorescence-based result. While it is known that the strain used produces predominantly lactic acid (>90%) during growth on lactose, low levels of acetic acid production might lead to slight misestimation of the lactic acid production level. The switch to heterofermentative metabolism (acetic and lactic acid formation) is known to occur in conditions with reduced glycolytic flux (Thomas et al., 1979, 1980) or increased exposure to molecular oxygen and intracellular redox balance (Garrigues et al., 1997; Lopez de Felipe et al., 1998; Melchiorsen et al., 2000), which could both be apparent during the prolonged assay developed here. Therefore, we investigated whether the induced non-growing state led to changes in fermentation pathways. Under the experimental conditions used, the cells remained a homofermentative (>90% of carbon flux toward lactic acid) metabolite profile during the 2-week incubation (Figure 2D). The final concentrations of lactic acid determined reached almost 20 mM, whereas concentrations of formic and acetic acid did not exceed 1 mM (Figure 2D). Moreover, the lactic acid concentrations determined matched accurately (R-squared value of 0.94) with the concentrations estimated based on fluorescence measurements using the lactic acid titration curve (Figure 2C).

### Sugar and Arginine Utilization Are Strain and Pre-culture Dependent

**FIGURE 3:**
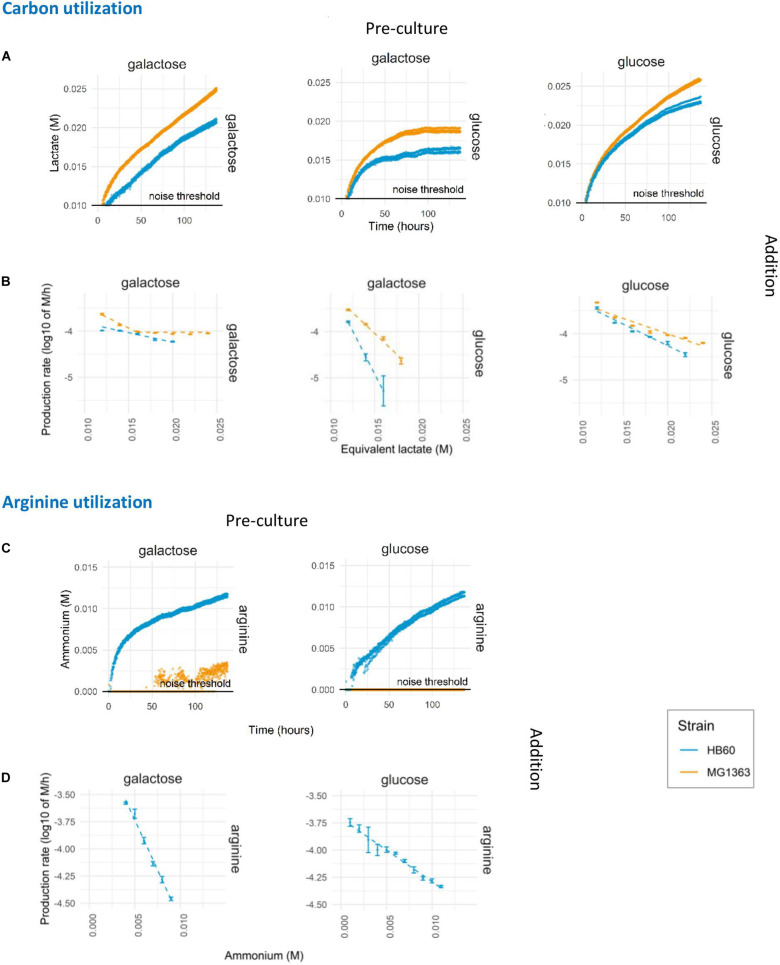
Prolonged assay of carbon and arginine utilization. *L. lactis* MG1363 (orange) and HB60 (blue) were harvested at mid-exponential growth following pre-culture (top label) in 1% w/v of glucose or galactose and subsequently transferred to translationally blocked assay mediums with different carbon source (right side label) at 2.0E+07/mL cell density. **(A)** Lactate production. **(B)** The decline in lactate production rates. **(C)** Ammonium production. **(D)** The decline in ammonium production rates. Dashed lines indicate the linear fits of the data. Noise threshold was determined from the non-biological fluctuation of signal based on a negative control at pH 6.5 (lactate production) and pH 5.5 (ammonium production). Error bars indicate standard deviation of the experiment (*n* = 4).

The presented standardized protocol allows for the testing of numerous modulations of environmental parameters as well as the comparison of different strains. To demonstrate this, the developed protocol was used to test the influence of pre-culture conditions on the long-term acidification activity using different pre-culture and assay substrates. In addition, the wild-type strain *L. lactis* MG1363 (plasmid-free derived of NCDO712) and its experimentally evolved derivative HB60 (Bachmann et al., 2013) were employed to further characterize the phenotype difference of these strains. Expression for carbon source utilization pathways is governed by carbon catabolite repression to ensure hierarchical utilization of preferred carbon source. As a consequence of catabolite repression, it was not unexpected to find that mid-exponential *L. lactis* MG1363 and HB60 pre-cultured in glucose showed no detectable utilization of galactose when it was provided to translationally blocked cells in the assay medium (data not shown). Growth on glucose is known to effectively repress the expression of the enzymes of the Leloir pathway that is required to import and utilize galactose as a carbon and energy source. In contrast, galactose pre-cultured *L. lactis* could effectively utilize either glucose or galactose during the translationally blocked assay conditions (Figure 3A middle and left). However, the glucose utilization rate (as measured by lactic acid formation rate) rapidly declined and halted within 75 h (Figure 3B middle). Interestingly, we observed higher acidification rates in MG1363 than HB60, which is in agreement with previous findings where due to a point-mutation (F65L) in *ptnD*, HB60 displays reduced glucose import activity and consequently a lower glycolytic flux compared to its parental strain (Bachmann et al., 2013). Higher acidification rates were also observed for galactose pre-cultured MG1363 in the prolonged assay in comparison to galactose pre-cultured HB60, irrespective of the carbon source provided during the assay (glucose or galactose). Notably, the acidification rates seem to decline faster in HB60 as compared to MG1363 (Figure 3B) under all assay conditions. Within 5 days of measurement, up to 25-fold decrease in acidification rates was found for strain HB60 as opposed to roughly 20-fold for strain MG1363. Since *L. lactis* MG1363 grown on galactose and HB60 displays a mixed-acid fermentation profile, it may be that the calculated acid production is somewhat misestimated, but the kinetics of production decline remains reliable due to normalization to the produced amount of acid. Moreover, the pH values during the prolonged acidification assay are between 5.75 and 6.5, where both acetic (pKa = 4.75) and formic (pKa = 3.75) acid are predominantly present in their deprotonated form (>90 and >99%, respectively). The calculated proton equivalent is barely affected by undissociated H^+^ ions from formic acid, but rather slightly affected by undissociated H^+^ ions from acetic acid. In our setup, a higher flux toward acetate would potentially result in more protons released due to the stoichiometry of the end products and the high buffer pH which ensures that lactate and acetate are deprotonated. This means our system might underestimate the differences in acidification rate between MG1363 and HB60 and the discussed effect might actually be larger than what is shown.

Next to lowering the pH by carbon fermentation and organic acid production, the presented method also allows for similar measurements of pathways that lead to metabolite production that increase the environmental pH. To exemplify this possibility, we characterized the arginine utilization pathway activity over extended periods of time in *L. lactis* strains MG1363 and HB60 that were incubated in assay media that lack a carbon source, and with an initial pH set to 5.5. This enables the detection of a pH increase due to the formation of one of the arginine pathway products, ammonium (Figure 3C). Besides ammonium, arginine catabolism by the arginine deiminase (ADI) pathway also generates ornithine, CO_2_ and ATP (Poolman et al., 1987), which contribute to the enhanced acid stress tolerance observed in cells that actively express this pathway (Budin-Verneuil et al., 2004). The detected pH-increase curves established that there is significant ammonium production in *L. lactis* HB60, but that this activity could not readily be detected in MG1363 (Figure 3C). Especially glucose pre-cultured cells of MG1363 appeared to lack ammonium production entirely, whereas the very modest pH increase observed in galactose pre-cultured MG1363 cells suggests that these cells did convert arginine to ammonium, albeit at a marginal and barely detectable level. Strikingly, when analyzing the ammonium production rates decline over time in *L. lactis* HB60 (Figure 3D), we observed a faster decline in galactose pre-cultured cells compared to glucose pre-cultured cells. Overall, this demonstrates the suitability and sensitivity of the method to capture the metabolic pathway activity levels over prolonged periods of time, and it enables the study of strain and pre-culture dependent differences.

## Discussion

Here we describe a high-throughput approach and analysis pipeline for prolonged measurements of metabolic activity using translationally blocked *L. lactis* cells. As model pathways, we employed carbohydrate and amino acid utilization of which the products can be readily detected by measurement of the pH of the medium. The novelty in the presented approach lies in the fact that real-time monitoring of lactic acid formation or ammonium accumulation due to arginine deamination can be performed for up to several weeks by a relatively straightforward and simple microplate-based assay. Prolonged product-formation measurements can be readily tracked in low-density, translation-blocked cells, which is critical to prevent cell growth and adaptation and product inhibition. To some extent, these translationally blocked cells may also mimic aspects of cellular physiology and pathway persistence in the non-growing state that many cells experience during environmental and industrial conditions. This possible parallel opens several avenues to test industrially and environmentally interesting phenotypic properties in changing environments. In the case that strains of interest carry erythromycin resistance cassettes, there are multiple antibiotic alternatives that could likely be employed for the same purpose, such as chloramphenicol, and azithromycin (Svetlov et al., 2017; Yerlikaya, 2019). Alternatively, a non-growing state can be achieved by nitrogen starvation or omission of essential nutrients, although in such a system the complete inhibition of translation cannot be guaranteed as internal turnover of the limiting molecules might occur.

The presented method was used to compare the metabolic capacity of two closely related strains. *L. lactis* MG1363 and HB60 which is a derivative of MG1363 that contains a point-mutation (F65L) in its *ptnD* gene. HB60 is reported to have a higher ATP yield when growing on glucose at the expense of its growth rate (Bachmann et al., 2013). However, the expression of other ATP-generating pathways, such as arginine deiminase pathway was not investigated in this strain, to date. While the mutated phosphotransferase system (PTS^Man^) serves mainly as a high-affinity glucose transport system in *L. lactis*, it has also been suggested to be involved in galactose transport in *L. lactis* MG1363 (Neves et al., 2010). Investigating carbon and arginine utilization, we demonstrated that acidification rates of MG1363 were higher than those of HB60 not only for glucose utilization, but also for galactose utilization. Interestingly, we also observed significantly higher ammonium production through arginine deimination in translationally blocked cells of strain HB60 than MG1363. While the amount of arginine in the initial study with this strain was relatively low, the catabolization of arginine in HB60 could potentially contribute to the observed increased biomass yield. It has been reported that arginine utilization during active growth could increase biomass yield up to 25% in another *L. lactis* strain (Palmfeldt et al., 2004). The expression of the arginine deiminase pathway has been linked to carbon catabolite repression (Gaudu et al., 2003; Zomer et al., 2007). The lower growth rate of HB60 might possibly play a role in relieving repression of this pathway either directly through interaction of CcpA with the *cre* elements in the *arc* operon or indirectly through, e.g., FBP levels which are expected to be lower in the slow growing HB60 (Bachmann et al., 2013). In chemostat cultures, it was observed that arginine consumption increases up to a dilution rate of 0.5 h^–1^ above which it rapidly drops again (Goel et al., 2015). Based on this data it seems plausible that a reduction of the maximum growth rate can result in a higher arginine utilization, indicating that exploration of this activity in strains that are known to display reduced growth rates e.g., *ldh* or *ccpA* mutants (Platteeuw et al., 1995; Luesink et al., 1998; Bongers et al., 2003) would be of interest.

Next to the ability of the assay to measure pathway activities, we also observed differences in production rate decline in relation to pre-culture conditions or strains. A prominent example was the glucose-utilization assay of galactose- pre-cultured cells which showed rapid acidification decline and complete halt within 100 h of measurement. The underlying mechanism that caused acidification to stop after the transition to the preferred carbon source glucose remains to be deciphered, but it has been suggested that large overshoots in intracellular metabolites can be toxic due to osmotic and hydration effects (Korem Kohanim et al., 2018). Such activity decline could potentially be associated with changes in the physiological state of the population over time, including rate of cellular viability and membrane integrity loss. Detailed analysis of such underlying effects, may reveal heterogeneity in the bacterial population, including the potential presence of persister subpopulations in cultures produced under different pre-culture conditions that may explain the (bi-phasic) decline in metabolic activities during the subsequent assay conditions. The current approach is a valuable addition toward answering fundamental questions on catalytic stability and cellular fitness, particularly in non-growing environmental conditions.

The presented method provides additional insight on the complete pathway activity of intact cells. While omics analysis produces substantial information on composition and level of transcripts and proteins in response to variation in a specific growth condition, they have a reduced throughput and are not necessarily able to distinguish between active and non-active proteins/pathways. The combination of omics technologies with extensive physiological measurement contributes to our understanding of cellular performance during long-term incubations under non-growing conditions. In addition, determination of the specific activity level of relevant enzymes or pathways in cells harvested from the assay conditions could be used to determine enzyme decay. The simplicity of the method developed is very attractive, while it does not compromise on the level of assay condition definition. Consequently, omission, addition and dose titration of single components (e.g., metals, vitamins, amino acids, etc.) can be performed in a high throughput manner to decipher the effect of environmental conditions on the flux through certain pathways. Moreover, the influence of biochemical parameters on pathway activities, such as allosteric regulation, co-factor availability, pH, and temperature, can also be explored and related to its longevity. Although the present assay was on pathways that modulate the environmental pH (i.e., carbon flux and lactic acid production, and arginine utilization, and ammonium formation) that is monitored by a pH-dependent fluorescent reporter, one can envision the expansion to other metabolic pathways provided that product formation can be measured by fluorescence or other means of detection (e.g., luminescence and absorbance) that are compatible with high-throughput methodologies. Ultimately, this approach will allow to investigate the effect of environmental and genetic modulation on phenotypic properties and the optimization during prolonged catalysis in biotechnological applications, which is largely unexplored despite of its commercial interest.

## Data Availability Statement

All datasets generated for this study are included in the article/supplementary material.

## Author Contributions

ADWN executed the experimental work. All authors designed the study, participated in data interpretation, and wrote the manuscript, have read and approved the final manuscript.

## Conflict of Interest

The project was organized by and executed under the auspices of TiFN, a public—private partnership on pre-competitive research in food and nutrition. Funding for this research was obtained from Friesland Campina (Wageningen, Netherlands), CSK Food Enrichment (Wageningen, Netherlands) and the Top-sector Agri and Food. The public partners were responsible for the study design, data collection and analysis, decision to publish, and preparation of the manuscript. The private partners have contributed to the project through regular discussion. HB was employed by NIZO Food Research. The remaining authors declare that the research was conducted in the absence of any commercial or financial relationships that could be construed as a potential conflict of interest.
